# The Effect of Vitamin D Deficiency as a Risk Factor of Early Fragmentation in Legg-Calve-Perthes Disease: A Prospective Study

**DOI:** 10.7759/cureus.57274

**Published:** 2024-03-30

**Authors:** Syed Faisal Afaque, Vikas Verma, Udit Agrawal, Suresh Chand, Vaibhav Singh, Ajai Singh

**Affiliations:** 1 Department of Pediatric Orthopedics, King George's Medical University, Lucknow, IND; 2 Department of Orthopedics, All India Institute of Medical Sciences Bhopal, Bhopal, IND

**Keywords:** legg-calve-perthes disease (lcpd), re-ossification stage, vitamin d, risk factor, fragmentation stage

## Abstract

Introduction: Legg-Calve-Perthes disease (LCPD) is a disorder involving the hips in young children of preschool and school-going age groups, more common in 4-8 years. The insufficient blood supply to the femoral head is the main reason behind various etiologic theories. Multiple factors affect the natural progression of the disease. The natural progression of the disease involves early avascular necrosis, fragmentation, reconstitution, and healed stages. In the fragmentation stage, the bony epiphysis begins to fragment, and the subchondral radiolucent zone (crescent sign) is the result of a subchondral stress fracture, which later on determines the extent of a necrotic fragment of the femoral head. These changes later contribute to changes in the shape of the femur head and the extent of deformity. As vitamin D plays a vital role in the onset of the fragmentation stage, we conducted a study to assess the effect of vitamin D deficiency as a risk factor for early fragmentation in Legg-Calve-Perthes disease.

Methods: In our study, 50 patients aged 4-12 years were examined over three years and classified according to Catterall and Herring's lateral pillar classification; the length of the fragmentation stage and the vitamin D level were considered. A vitamin D level of less than 20 ng/mL was labeled as the deficient group, 20-30 ng/mL as the insufficient group, and more than 30 ng/mL as the sufficient (normal) group.

Results: The critical fragmentation stage was significantly longer (more than 12 months) in vitamin D deficiency (34%), leading to a higher risk of deformity and extrusion of the femoral head, which led to higher rates of surgical intervention and containment procedures.

Conclusion: The fragmentation stage is critical in the course of LCPD. Vitamin D levels play a vital role in predicting the prognostic of LCPD, and it should be measured in all patients of LCPD. Patients with normal vitamin D levels have a comparatively shorter fragmentation stage duration than patients with insufficient or deficient levels, leading to a lesser duration of femoral head damage.

## Introduction

Legg-Calve-Perthes disease (LCPD) can occur in children of any age group, but it mostly affects preschool and school-going age groups, typically between four and eight years of age [1]. It is more common in males [2], and bilateral cases have been reported up to 10% [3]. Several associations exist between various etiologic factors, but no definite relationship has been established. It is still unclear what causes the vascular compromise leading to the disease's clinical manifestation. Burwell et al. reported various anthropomorphic abnormalities and a delay of skeletal maturation, which have a higher incidence of developing the disease [4]. Some of the factors associated with the disease are reduced levels of protein C and protein S [5] and active and passive smoking [6], which have also been found to be associated with the disease [7]. Transient synovitis is found to be a precursor of the disease, and such children are more prone to develop LCPD [8]. A previous history of trauma also contributes to the causation of the disease. A prior history of trauma before the early symptoms has been found in many children [9]. The primary etiology is unknown; the vascular compromise of the femoral head is universally accepted.

The natural progression of the disease includes stages of avascular necrosis, fragmentation, re-ossification, and healing stages. The deformation of the femoral head results from the structural failure of the bony trabeculae of the femoral epiphysis under normal stress of weight-bearing and muscular activity [10]. During the fragmentation stage, the femoral head is most vulnerable to deformation. The identification of the early fragmentation stage is of prognostic value. The extrusion of the femoral head mainly contributes to head deformation, which mostly occurs during the late fragmentation stage. Therefore, any treatment provided during early fragmentation or before can help decrease the amount of femoral head deformation. Treatment instituted at a late stage of fragmentation (stage 3B) or after that is only remedial/salvage in nature [11]. Various prognostic factors determine the course of the disease [12]. The most important are the residual deformity of the femoral head and the degree of hip joint congruency. Vitamin D is essential for bone mineralization. It is a well-established fact that vitamin D deficiency leads to decreased calcium absorption, which causes the weakening of bone architecture [13].

The literature review revealed only one study suggesting the role of vitamin D as a prognostic factor for the fragmentation stage [14]. However, that study did not discuss the role of vitamin D supplementation before the stage of fragmentation sets in. In this study, we aimed to assess the effect of vitamin D on the course of LCPD, particularly on the fragmentation and re-ossification stages.

## Materials and methods

This study was a prospective cohort study of patients with LCPD aged 4-12 years who came to the Department of Pediatric Orthopedics outdoor facility, King George's Medical University, Lucknow, from December 2019 to November 2022. The Institutional Ethics Committee of King George's Medical University reviewed and approved the study's protocol.

The inclusion criteria were patients aged 4-12 years having unilateral LCPD with no treatment or intervention done in the past six months. Patients with stages 2 and 3A were included. The exclusion criteria were patients having bilateral disease, patients of stage 3B onward (late re-ossification), patients with concomitant rickets, patients with a history of previous bisphosphonate or vitamin D therapy, patients with a history of prior surgical intervention, patients with any associated lesions of the proximal femur (neoplastic), and patients with any metabolic disorder or any related syndromes.

A total of 50 patients who fulfilled the inclusion and exclusion criteria were enrolled in the study and screened. X-ray of the pelvis with both hip anteroposterior and frog lateral views was taken at the time of presentation along with the levels of vitamin D. The X-ray staging of the disease was done using Catterall and Herring's lateral pillar classification. Parents were then counseled about the standard treatment protocol and gave consent. The data of clinical signs and symptoms and laboratory examinations were documented and registered for each patient and analyzed descriptively. Patients were divided into three groups of vitamin D based on the quantitative measurement of the serum levels of 25-hydroxyvitamin D (25(OH)D). Values of less than 20 ng/mL were considered deficient, 20-30 ng/mL insufficient, and 30-40 ng/mL optimal sufficient. Patients were regularly followed up, and their stage of the disease was noted.

The sample size was calculated using the formula based on the prevalence. Data were collected in a pre-defined proforma. It was then entered into a Microsoft Excel spreadsheet (Microsoft Corp., Redmond, WA) and analyzed using the Statistical Package for Social Sciences (SPSS) version 25.0 (IBM SPSS Statistics, Armonk, NY). Descriptive statistics were first deduced, and the variables were categorized into continuous and categorical. Continuous variables were further categorized into parametric and nonparametric using histogram plots. Pearson's chi-square test, Pearson's correlation test, and post hoc analysis were performed using the Bonferroni test to interpret the results.

## Results

**Figure 1:**
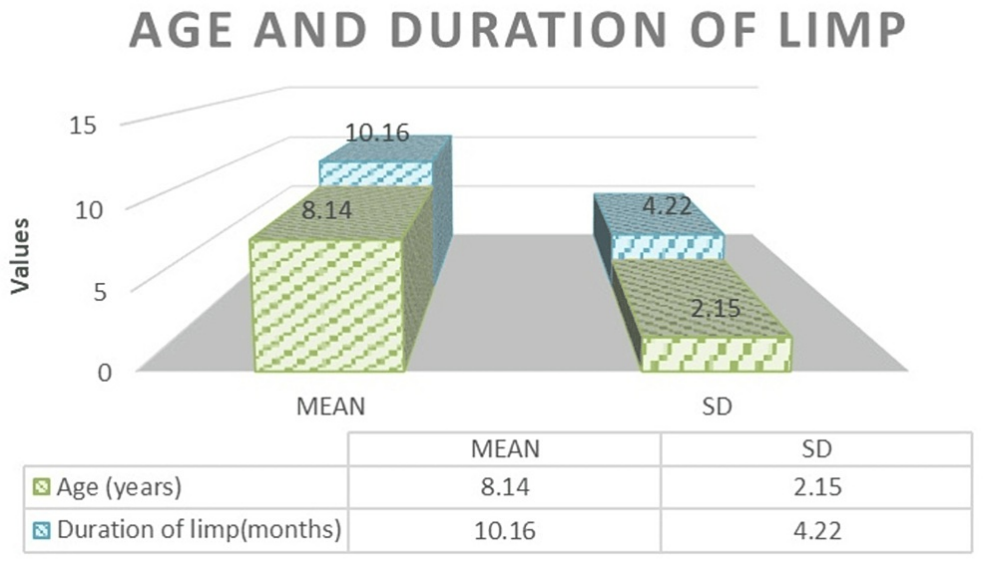
Distribution of age and duration of limp

This study included 50 patients fulfilling inclusion criteria, with a mean age of 8.14±2.15 years and a mean duration of limp of 10.16±4.22 months (Figure 1). Among them, 58% were females, and 42% were males, with predominantly right-side (54%) involvement.

**Figure 2:**
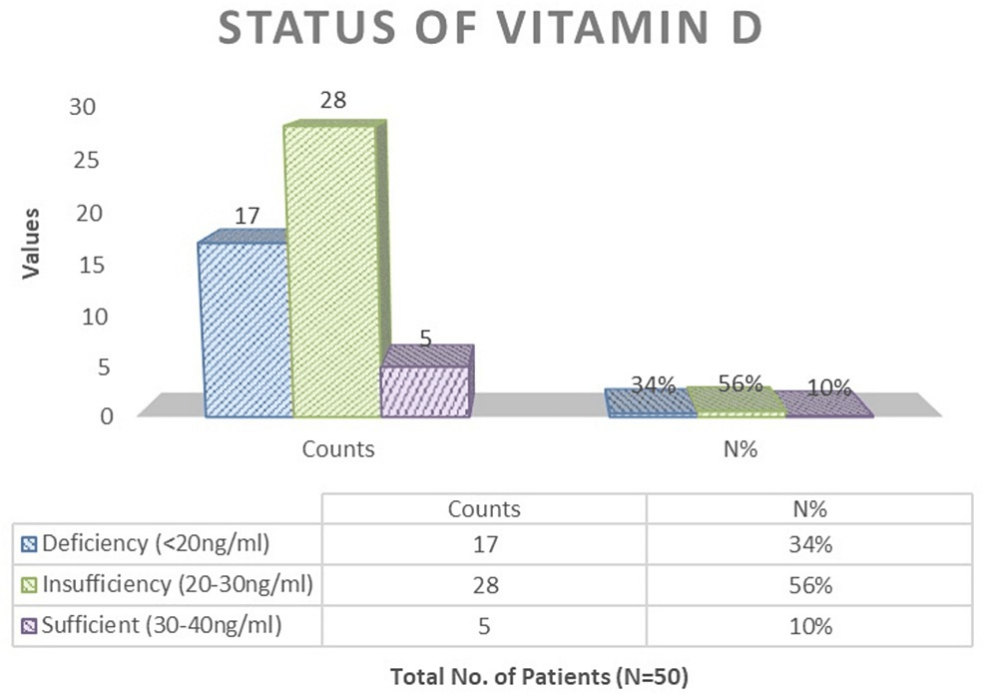
Status of vitamin D among the patients

Vitamin D deficiency (<20 ng/mL) was seen in 34% of children, 56% with insufficient vitamin D (20-30 ng/mL), and 10% with sufficient vitamin D levels (30-40 ng/mL) (Figure 2).

In the present study, there was no significant difference in the distribution of vitamin D status between the genders (Table 1) (p>0.05).

**Table 1:** Status of vitamin D (ng/mL) concerning gender among the patients

Vitamin D status	Female		Male	
	Count	N%	Count	N%
Deficient (<20 ng/mL)	8	27.6%	9	42.9%
Insufficient (20-30 ng/mL)	17	58.6%	11	52.4%
Sufficient (30-40 ng/mL)	4	13.8%	1	4.8%

A comparison of the mean duration level of the fragmentation and re-ossification stages with vitamin D status among the participants, with a p-value of <0.05, was considered statistically significant. The post hoc analysis was performed using the Bonferroni test for the inter-group comparison of the mean difference.

**Figure 3:**
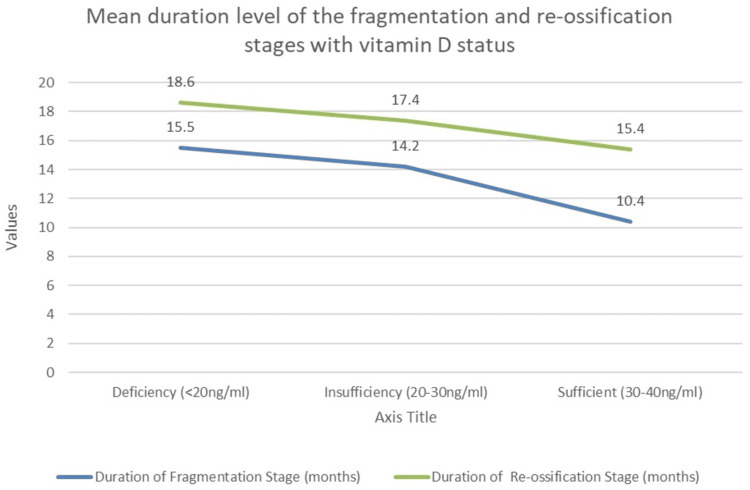
Comparison of the mean duration level of the fragmentation and re-ossification stages with vitamin D status among the participants

On the assessment of vitamin D status with the duration of fragmentation stage, there was significantly longer duration in the fragmentation stage in the vitamin D-deficient group (15.5±1.3) compared to the vitamin D-insufficient (14.2±1.9) and vitamin D-sufficient groups (10.4±3.1) (p<0.01). Similarly, on the comparison of vitamin D status with a duration of the re-ossification stage, there was a significantly longer duration in a re-ossification stage in the vitamin D-deficient group (18.6±1.0) compared to the vitamin D-insufficient (17.4±1.6) and vitamin D-sufficient groups (15.4±2.1) (p<0.01) (Table 2) (Figure 3).

**Table 2:** A comparison of the mean duration level (months) of the fragmentation and re-ossification stages with vitamin D status (ng/mL) among the participants ANOVA: analysis of variance

Vitamin D status							ANOVA test
Duration of stage (months)	Deficient (<20 ng/mL)		Insufficient (20-30 ng/mL)		Sufficient (30-40 ng/mL)		
	Mean	SD	Mean	SD	Mean	SD	p-value
Fragmentation	15.5	1.3	14.2	1.9	10.4	3.1	0.01
Re-ossification	18.6	1.0	17.4	1.6	15.4	2.1	0.01

There was no significant difference in the vitamin D status of the patients at presentation according to their X-ray staging (Table 3) (p>0.05).

**Table 3:** Comparison of the vitamin D status (ng/mL) to X-ray staging at presentation

Vitamin D status	X-ray staging at presentation			
	2A		2B	
	Count	N%	Count	N%
Deficient (<20 ng/mL)	16	34.0%	1	33.3%
Insufficient (20-30 ng/mL)	26	55.3%	2	66.7%
Sufficient (30-40 ng/mL)	5	10.6%	0	0%

**Figure 4:**
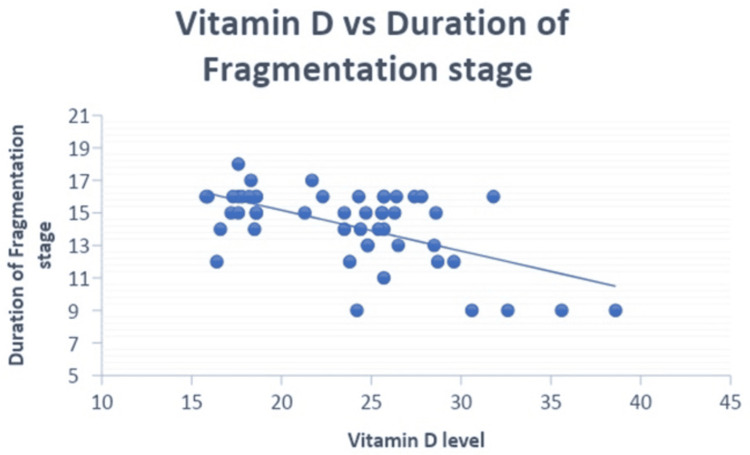
Negative correlation of vitamin D level (ng/mL) with the duration of the fragmentation stage

**Figure 5:**
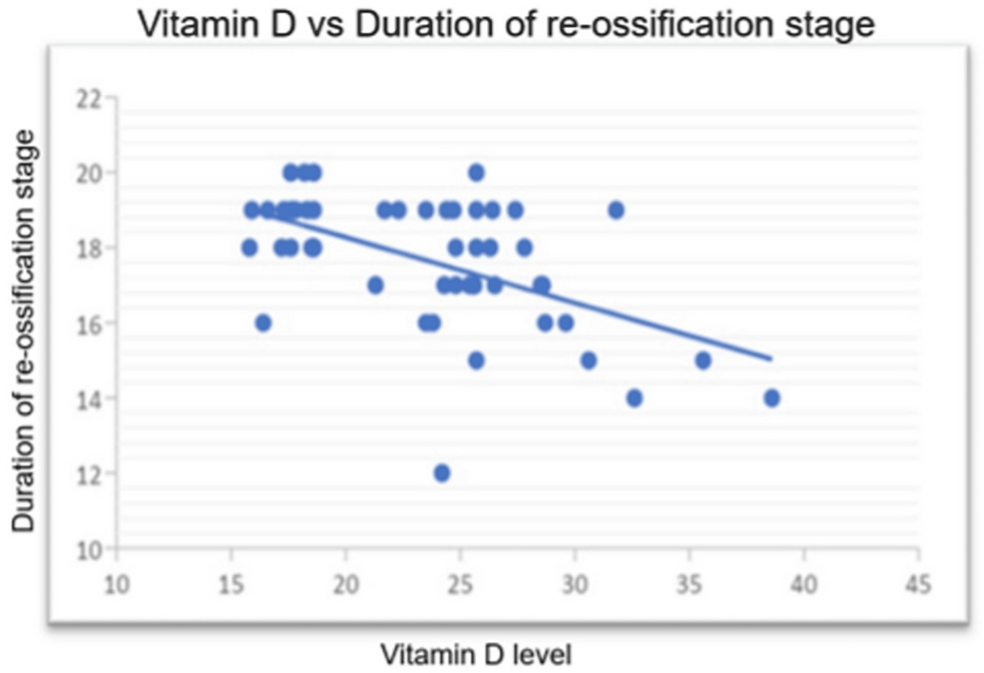
Negative correlation of vitamin D level (ng/mL) with the duration of the fragmentation stage

There was a significant negative correlation of vitamin D level with the duration of the fragmentation stage (Figure 4) (r=-0.585; p<0.01) and the duration of the re-ossification stage (Figure 5) (r=-0.535; p<0.01) among patients. There was no significant correlation between the vitamin D level and the duration of limp among the patients (Table 4).

**Table 4:** Pearson's correlation of vitamin D level with the duration of limp, fragmentation stage, and re-ossification stage among the study participants

Duration (months)		Serum vitamin D (ng/mL), at presentation
Duration of limp	r	-0.103
	p-value	0.476
Duration of fragmentation	r	-0.585
	p-value	0.01
Duration of re-ossification	r	-0.535
	p-value	0.01

Significant limb shortening was seen in children with vitamin D deficiency compared to those with insufficiency and normal vitamin D levels (Table 5).

**Table 5:** The mean level of shortening of the limb (cm) with vitamin D status (ng/mL) among the participants

Vitamin D status	Shortening of the limb (cm)		p-value
	Mean	SD	
Deficient (<20 ng/mL)	1.70	0.57	0.05
Insufficient (20-30 ng/mL)	0.83	0.29	-
Sufficient (30-40 ng/mL)	-	-	-

## Discussion

LCPD is a disease of unknown etiology, though the identification of prognostic factors dramatically affects the natural course of the disease [14]. The natural history of the disease includes stages of avascular necrosis, fragmentation, re-ossification, and healing [1]. The damage to the femoral head mostly occurred during the fragmentation stage. Therefore, the identification of this stage is of prognostic value [2]. In our study, the mean age of presentation was 8.14±4.22 years, consistent with the literature [1] and more common in girls [2]. The vitamin D level was deficient in 34% and insufficient in 56%, which is quite common among the Indian population [13].

On the assessment of vitamin D status with a duration of fragmentation stage, there was a significantly longer duration in the fragmentation stage in the vitamin D-deficient group (15.5±1.3) compared to vitamin D-insufficient (14.2±1.9) and vitamin D-sufficient groups (10.4±3.1) (p<0.01), which is consistent with the study conducted by Al-Naser et al. [12]. On the comparison of vitamin D status with the duration of the re-ossification stage, there was a slightly longer duration noted in a re-ossification stage in the vitamin D-deficient group (18.6±1.0) compared to the vitamin D-insufficient (17.4±1.6) and vitamin D-sufficient groups (15.4±2.1) (p<0.01); similar results were shown by Al-Naser et al. [12] in their study. Vitamin D has a beneficial effect on bone health; it has been found to increase bone mineral density, as seen in previously conducted studies by Karimian et al. [15] and Laird et al. [16]. It also reduces the impact of pro-inflammatory cytokines on the bone [14]. Thus, supplementation is necessary, especially in LCPD, where determining the prognostic stage affects the residual deformity of the femoral head. Vitamin D levels in children depend on various socioeconomic and personal factors [16]. Therefore, 40-70 ng/mL supplementation is required for optimal health benefits [13,17].

The limitations of our study include the level of vitamin D at each follow-up not being taken into account, the longer duration of the study, and the lack of including other associated factors at subsequent follow-up, such as activities of daily living and the nutritional status of the child.

## Conclusions

The fragmentation stage is critical in the course of LCPD. Any intervention done until this stage has prognostic value in the natural history of the disease. Therefore, determining the factors affecting this stage is necessary. Levels of vitamin D play a vital role in predicting the prognosis of the disease, and it should be measured in all patients of LCPD. Patients with normal vitamin D levels have a comparatively shorter fragmentation stage duration than patients with insufficient or deficient levels, leading to a lesser duration of femoral head damage. Hence, vitamin D should be prescribed in such groups of patients. However, more such studies in a larger cohort are needed to validate the conclusion of this study.
